# Effect of Mesenchymal Stem Cells Overexpressing BMP-9 Primed with Hypoxia on BMP Targets, Osteoblast Differentiation and Bone Repair

**DOI:** 10.3390/biology12081147

**Published:** 2023-08-19

**Authors:** Jessica Emanuella Rocha Moura Paz, Leticia Faustino Adolpho, Jaqueline Isadora Reis Ramos, Rayana Longo Bighetti-Trevisan, Robson Diego Calixto, Fabiola Singaretti Oliveira, Adriana Luisa Gonçalves Almeida, Marcio Mateus Beloti, Adalberto Luiz Rosa

**Affiliations:** Bone Research Lab, School of Dentistry of Ribeirão Preto, University of São Paulo, Avenida do Café, s/n, Ribeirão Preto 14040-904, SP, Brazil; jessicamourapaz@usp.br (J.E.R.M.P.); leticia.adolpho@usp.br (L.F.A.); jaqueline.isadora.ramos@usp.br (J.I.R.R.); rayana.bighetti@usp.br (R.L.B.-T.); robsonzahaila@usp.br (R.D.C.); oliveira@forp.usp.br (F.S.O.); aalmeida@forp.usp.br (A.L.G.A.); mmbeloti@usp.br (M.M.B.)

**Keywords:** BMP-9, bone, cell therapy, HIF-1α, hypoxia, stem cells

## Abstract

**Simple Summary:**

Bone formation is regulated by proteins such as bone morphogenetic proteins (BMPs) as well as by oxygen. We previously demonstrated that cell therapy using stem cells genetically modified to express BMP-9 enhances bone formation in skull defects. Here, it was evaluated the effect of these cells submitted to low oxygen tension (hypoxia) on bone cell (osteoblast) differentiation and bone repair. The effect of stem cells expressing BMP-9 on osteoblast differentiation was evaluated using the solution where these cells were grown (conditioned medium). The bone formation induced by stem cells expressing BMP-9 directly injected into rat skull defects was also evaluated. The results demonstrated that the conditioned medium generated under hypoxia favored osteoblast differentiation. Hypoxia-conditioned stem cells expressing BMP-9 did not increase bone repair compared with stem cells expressing BMP-9 under normoxia. Thus, despite the lack of effect of hypoxia on bone formation, the enhancement of osteoblast differentiation can drive further investigations on the regulation of BMP-9 and oxygen availability in the context of therapies to induce bone regeneration, which may improve the clinical treatment of large bone fractures and defects.

**Abstract:**

Bone formation is driven by many signaling molecules including bone morphogenetic protein 9 (BMP-9) and hypoxia-inducible factor 1-alpha (HIF-1α). We demonstrated that cell therapy using mesenchymal stem cells (MSCs) overexpressing BMP-9 (MSCs^+BMP-9^) enhances bone formation in calvarial defects. Here, the effect of hypoxia on BMP components and targets of MSCs^+BMP-9^ and of these hypoxia-primed cells on osteoblast differentiation and bone repair was evaluated. Hypoxia was induced with cobalt chloride (CoCl_2_) in MSCs^+BMP-9^, and the expression of BMP components and targets was evaluated. The paracrine effects of hypoxia-primed MSCs^+BMP-9^ on cell viability and migration and osteoblast differentiation were evaluated using conditioned medium. The bone formation induced by hypoxia-primed MSCs^+BMP-9^ directly injected into rat calvarial defects was also evaluated. The results demonstrated that hypoxia regulated BMP components and targets without affecting BMP-9 amount and that the conditioned medium generated under hypoxia favored cell migration and osteoblast differentiation. Hypoxia-primed MSCs^+BMP-9^ did not increase bone repair compared with control MSCs^+BMP-9^. Thus, despite the lack of effect of hypoxia on bone formation, the enhancement of cell migration and osteoblast differentiation opens windows for further investigations on approaches to modulate the BMP-9-HIF-1α circuit in the context of cell-based therapies to induce bone regeneration.

## 1. Introduction

Mesenchymal stem cells (MSCs) are undifferentiated cells derived from tissues such as bone marrow and fat, characterized by their ability to self-renew and differentiate into adipocytes, chondrocytes and osteoblasts [1]. The bone matrix is synthesized by osteoblasts, which are of great interest in cell-based therapies focused on the treatment of challenging bone defects [2,3,4]. The differentiation of MSCs into osteoblasts is a complex phenomenon orchestrated by a plethora of cell signaling pathways, including those triggered by bone morphogenetic proteins (BMPs) [5,6,7].

BMPs are signaling molecules belonging to the transforming growth factor beta superfamily that are involved in embryogenesis, and some of them are potent osseoinductor factors [8,9,10]. The BMPs bind to serine/threonine kinase receptors and transduce signals through SMAD family member (Smad)-dependent and non-Smad-dependent mechanisms, both involved in osteoblast differentiation [8,11]. The most osteogenic BMPs are BMP-2, -4, -6, -7 and -9, and several studies have shown that they induce osteoblast differentiation and bone formation [12,13,14,15]. By using recombinant adenovirus-mediated gene delivery to overexpress 14 types of BMPs (BMP-2 to -15) in C2C12 cells, it was demonstrated that BMP-6 and -9 induced higher alkaline phosphatase (ALP) activity and generated the most robust and mature ectopic ossification when implanted into the quadriceps of athymic mice [16]. Additionally, it was observed that BMP-3, a negative regulator of bone formation, inhibited the ossification induced by BMP-2, -6 and -7 but not BMP-9, suggesting that BMP-9 triggers different cellular mechanisms to promote osteogenesis [16]. One of these mechanisms involves Wnt, as the participation of the Wnt/β-catenin signaling pathway in the osteoblast differentiation of MSCs induced by BMP-9 was observed [17,18]. BMP-9 promoted bone formation and crystallinity as compared to BMP-2 in rat calvarial defects injected with chitosan microparticles coated with BMPs [19]. We demonstrated that MC3T3-E1 cells grown on a nanostructured surface were more sensitive to the positive effects of exogenous BMP-9 on osteoblast differentiation [20]. Furthermore, genetically edited MSCs to overexpress BMP-9 (MSCs^+BMP-9^) using clustered regularly interspaced short palindromic repeats/associated nuclease Cas-9 (CRISPR–Cas-9) exhibited high in vitro osteogenic potential and enhanced bone repair in rat calvarial defects [21].

To keep the physiological functions of cells and tissue, the bone needs an adequate concentration of oxygen, and when this concentration is reduced, a hypoxic condition, the bone health is affected [22]. Indeed, hypoxia regulates bone regeneration, and hypoxia-inducible factors (HIFs) that contain two heterodimeric transcription factors, HIF-α and HIF-β, are the main players in this tissue response [23]. Under hypoxic conditions, HIF-α binds to HIF-β, translocates into the nucleus and binds to the hypoxia response element to induce the transcription of hypoxia-related genes [24]. Among HIFs, HIF-1α regulates bone development and regeneration by acting on angiogenic–osteogenic coupling and on osteoblast metabolic pathways [23,25,26]. The BMP-9 increased the HIF-1α protein expression and the inactivation of HIF-1α with either a chemical inhibitor or siRNA reduced the extracellular matrix mineralization induced by BMP-9 in cultures of osteoblasts, indicating that HIF-1α plays an essential role in the BMP-9-mediated osteoblast differentiation [27]. Chromatin immunoprecipitation assays in immortalized mouse embryonic fibroblasts showed that the runt-related transcription factor 2 (Runx2) is a downstream target of HIF-1α that regulates osteoblast differentiation induced by BMP-9 [28].

Based on the enhanced bone formation induced by MSCs^+BMP-9^ and the role of the BMP-9-HIF-1α circuit on osteoblast differentiation, we hypothesized that hypoxia regulates the expression of the BMP signaling pathway components and targets of MSCs^+BMP-9^ and that these hypoxia-primed cells increase bone repair by affecting in a paracrine way the osteoblast differentiation of MSCs and osteoblasts, the main players in this process. To test this hypothesis, MSCs^+BMP-9^ were cultured with the hypoxia simulant cobalt chloride (CoCl_2_) that acts by preventing HIF-1α degradation [29]. We observed that hypoxia regulated BMP components and targets of MSCs^+BMP-9^ without affecting the amount of BMP-9. Additionally, the conditioned medium (CM) of hypoxia-primed MSCs^+BMP-9^ enhanced migration and the osteoblast differentiation of MSCs and MC3T3-E1 cells. We also demonstrated that hypoxia did not affect the bone formation induced by MSCs^+BMP-9^.

## 2. Materials and Methods

### 2.1. Hypoxia Induction and Duration of Its Effect on MSCs^+BMP-9^

#### 2.1.1. MSCs^+BMP-9^

MSCs derived from mouse bone marrow were previously immortalized, edited to overexpress BMP-9 using CRISPR–Cas-9 and characterized by our research group as described elsewhere [21]. These cells were cultured with different concentrations of CoCl_2_ (Sigma-Aldrich, St. Louis, MO, USA) and for different periods. Cell viability and HIF-1α protein expression were evaluated to determine the appropriate CoCl_2_ concentration and exposure time to induce hypoxia in MSCs^+BMP-9^. Additionally, the gene expression of the hypoxia targets, solute carrier family 2 (facilitated glucose transporter) member 1 (*Glut1*) and vascular endothelial growth factor A (*Vegfa*) was evaluated to confirm the hypoxia induction and to determine the lasting of its effect on MSCs^+BMP-9^ after CoCl_2_ removal.

#### 2.1.2. Analysis of the Cell Viability by Colorimetric Assay

MSCs^+BMP-9^ were seeded at a density of 2 × 10^4^ cells/well in 24-well culture plates (Corning Inc., Corning, NY, USA) and cultured in non-inducing culture medium composed by minimum essential medium, modification alpha (α-MEM, Gibco-Life Technologies, Grand Island, NY, USA), 20% fetal bovine serum (Gibco-Life Technologies), 100 U/mL penicillin (Gibco-Thermo Fisher Scientific, Waltham, MA, USA) and 100 µg/mL streptomycin (Gibco-Thermo Fisher Scientific) for 8 days. Twenty-four hours after cell seeding, the culture medium was changed to fresh medium, and after that, every 48 h. MSCs^+BMP-9^ were cultured either without (control) or with CoCl_2_ at the concentrations of 100, 150 and 200 μM during the last 3 days (from day 5 to 8), 5 days (from day 3 to 8) or 7 days (from day 1 to 8). On day 8, the cell viability was evaluated (n = 5) using a colorimetric assay with 3-(4,5-dimethylthiazol-2-yl)-2,5-diphenyltetrazolium bromide (MTT, Sigma-Aldrich) as described elsewhere [30]. The absorbance was measured at 570 nm using a μQuant spectrophotometer (BioTek Instruments Inc., Winooski, VT, USA). Based on these results, another set of experiments was performed to evaluate the viability of MSCs^+BMP-9^ cultured with CoCl_2_ at the concentrations of 10, 20, 40 and 50 μM.

#### 2.1.3. Analysis of the HIF-1α Protein Expression by Western Blot

MSCs^+BMP-9^ were seeded at a density of 10^6^ cells in 100 mm culture dishes (Corning Inc.) and cultured for 8 days. Based on the results of cell viability, MSCs^+BMP-9^ were cultured either without (control) or with CoCl_2_ at the concentrations of 40 and 50 μM during the last 3 and 5 days, and the HIF-1α protein was detected by using western blot following a conventional protocol. The proteins of each group were transferred to PVDF membranes (Bio-Rad Laboratories, Hercules, CA, USA), which were incubated with primary antibody anti-HIF-1α (1:1000; rabbit monoclonal antibody—14179S—Cell Signaling Technology, Danvers, MA, USA) and secondary antibody goat anti-rabbit IgG-HPR (1:3000—7074S—Cell Signaling Technology) overnight at 4 °C and 1 h at room temperature, respectively. Beta-actin protein was used as control and detected with primary antibody anti-beta-actin (1:1000; goat polyclonal antibody—sc1615—Santa Cruz Biotechnology, Dallas, TX, USA) and secondary antibody donkey anti-goat IgG-HPR (1:3000—sc2003—Santa Cruz Biotechnology). The protein bands were detected using Clarity^TM^ Western ECL Substrate (Bio-Rad Laboratories). HIF-1α was quantified (n = 3) using ImageJ Software (NIH, Bethesda, MD, USA) and normalized to beta-actin protein expression.

#### 2.1.4. Analysis of the *Glut1* and *Vegfa* Gene Expression by Real-Time Polymerase Chain Reaction (RT-qPCR)

MSCs^+BMP-9^ were seeded at a density of 10^6^ cells in 100 mm culture dishes (Corning Inc.) and cultured for 8 days. Based on the results of HIF-1α protein expression, MSCs^+BMP-9^ were cultured either without (control) or with CoCl_2_ at the concentration of 40 μM during the last 3 days. At the end of 3 days of CoCl_2_ treatment and 4, 8 and 12 h post-treatment, the *Glut1* and *Vegfa* gene expression was evaluated by using RT-qPCR following a conventional protocol. The samples were treated with Trizol reagent (Invitrogen, Carlsbad, CA, USA), and the total RNA was extracted using the SV Total RNA Isolation System kit (Promega, Fitchburg, WI, USA) and quantified using a NanoVue device (GE Healthcare, Chicago, IL, USA). The complementary DNA (cDNA), Fast SYBR Green Master Mix (Applied Biosystems, Foster City, CA, USA) and primer sequences (Applied Biosystems, Supporting Information, Table S1) were used in a QuantStudio™ 7 Flex System device for RT-qPCR (Applied Biosystems, Waltham, MA, USA). The reactions (n = 4) were performed, and the gene expressions were normalized to the endogenous control eukaryotic translation initiation factor 2B subunit 1 (alpha) (*Eif2b1*) using the 2^−ddCt^ method [31].

Based on the results of this section, the treatment with CoCl_2_ at the concentration of 40 μM during the last 3 days of an 8-day culture was selected to induce hypoxia in MSCs^+BMP-9^ and will be named hypoxia hereafter. Additionally, 4 h post-CoCl_2_ removal was selected as the time to collect the CM.

### 2.2. Effect of Hypoxia on the Expression of Components and Targets of the BMP Signaling Pathway in MSCs^+BMP-9^

#### 2.2.1. Analysis of the BMP-9 Protein Expression by Enzyme-Linked Immunosorbent Assay (ELISA)

MSCs^+BMP-9^ were seeded at a density of 10^6^ cells in 100 mm culture dishes (Corning Inc.) and cultured either under control or hypoxia condition. On day 8, cells were lysed, and the amount of BMP-9 was measured from the total protein using the mouse BMP-9 ELISA kit (Abcam, ab267576, Cambridge, UK) following the manufacturer’s instructions. The absorbance was measured at 450 nm using a μQuant spectrophotometer (BioTek Instruments Inc.), and the amount of BMP-9 (n = 3) was calculated using the manufacturers’ standard curve.

#### 2.2.2. Analysis of the Gene Expression of Components and Targets of the BMP Signaling Pathway by RT-qPCR

MSCs^+BMP-9^ were seeded at a density of 10^6^ cells in 100 mm culture dishes (Corning Inc.) and cultured either under control or hypoxia condition. On day 8, the gene expression of *Bmp-2*, *Bmp-4*, bone morphogenetic protein receptor, type 1A (*Bmpr1a*), *Smad1*, *Smad5*, hairy/enhancer-of-split related with YRPW motif 1 (*Hey1*) and *Runx2* was evaluated by using RT-qPCR. TaqMan Fast Advanced Master Mix (Applied Biosystems) and TaqMan probes (Applied Biosystems, Supporting Information, Table S1) were used to evaluate *Bmp-2* and *Bmp-4*, and Fast SYBR Green Master Mix (Applied Biosystems) and primer sequences (Applied Biosystems, Supporting Information, Table S1) were used to evaluate *Bmpr1a*, *Smad1*, *Smad5*, *Hey1* and *Runx2*. The reactions (n = 4) were performed, and the gene expressions were normalized to the endogenous control *Eif2b1* using the 2^−ddCt^ method [31].

### 2.3. Effect of CM of MSCs^+BMP-9^ Primed with Hypoxia on Cell Proliferation, Migration and Osteoblast Differentiation of MSCs and MC3T3-E1 Cells

#### 2.3.1. CM Production

MSCs^+BMP-9^ were seeded at a density of 10^6^ cells in 100 mm culture dishes (Corning Inc.) and cultured either under control or hypoxia condition. On day 8, the culture media were changed to serum-free α-MEM (Gibco-Life Technologies) supplemented with 100 U/mL penicillin (Gibco-Thermo Fisher Scientific) and 100 µg/mL streptomycin (Gibco-Thermo Fisher Scientific). After 4 h, CM of cells cultured under control (CM control) and hypoxia condition (CM hypoxia) were collected, centrifuged at 300× *g* and 4 °C for 30 min and stored at −80 °C.

#### 2.3.2. MSCs

MSCs were previously immortalized and characterized by our research group as described elsewhere [21]. These MSCs were seeded at a density of 2 × 10^4^ cells/well in 24-well culture plates (Corning Inc.), except cells for migration evaluation that were seeded at a density of 10^5^ cells/well in 6-well culture plates (Corning Inc.). They were cultured in either CM control or CM hypoxia both at the proportion of 1:1 with non-inducing culture medium, supplemented to a final concentration of 20% fetal bovine serum (Gibco-Life Technologies), except cultures for cell migration evaluation that were supplemented to a final concentration of 2% fetal bovine serum (Gibco-Life Technologies).

#### 2.3.3. MC3T3-E1 Cells

MC3T3-E1 cells, subclone 14 (ATCC, Manassas, VA, USA), were seeded at a cell density of 2 × 10^4^ cells/well in 24-well culture plates (Corning Inc.), except cells for migration evaluation that were seeded at a density of 10^5^ cells/well in 6-well culture plates (Corning Inc.). They were cultured either in CM control or CM hypoxia both at the proportion of 1:1 with osteogenic medium, which is non-inducing culture medium supplemented with 7 mM β-glycerophosphate (Sigma-Aldrich) and 5 μg/mL L-ascorbic acid (Sigma-Aldrich) and to a final concentration of 10% fetal bovine serum (Gibco-Life Technologies), except cultures for cell migration evaluation that were supplemented to a final concentration of 2% fetal bovine serum (Gibco-Life Technologies).

#### 2.3.4. Analysis of the Cell Proliferation by Colorimetric Assay

At 24, 48 and 72 h, cell proliferation was measured (n = 5) using MTT assay [30].

#### 2.3.5. Analysis of the Cell Migration by Scratch Method

Cell migration was evaluated using the scratch method [32]. After cell confluence, a scratch was made in the center of each well using a 100 µL pipette tip. At 6, 12 and 24 h, images were acquired using a digital camera Nikon DS-Fi1c (Nikon Instruments Inc., Melville, NY, USA) coupled to an inverted microscope Nikon Eclipse Ti-S (Nikon Instruments Inc.). The areas without cells (open areas) were delimitated and measured (n = 5) using the Nis Elements Br 5.02 software (Nikon Instruments Inc.).

#### 2.3.6. Analysis of the Gene Expression of Osteoblast Markers by RT-qPCR

MSCs were cultured in non-inducing culture medium and MC3T3-E1 cells in osteogenic medium for 5 days and in either CM control or CM hypoxia for the last 2 days. On day 7, Fast SYBR Green Master Mix (Applied Biosystems) and primer sequences (Applied Biosystems, Supporting Information, Table S1) were used to evaluate the gene expression of *Runx2*, osterix (*Sp7*), *Alp* and osteocalcin (*Oc*). The reactions (n = 4) were performed, and gene expression was normalized to the endogenous control *Eif2b1* using the 2^−ddCt^ method [31].

#### 2.3.7. Analysis of the RUNX2 and ALP Protein Expression by Western Blot

MSCs were cultured in non-inducing culture medium and MC3T3-E1 cells in osteogenic medium for 5 days and in either CM control or CM hypoxia for the last 2 days. On day 7, the western blots were performed using the primary antibodies anti-RUNX2 (1:1000; rabbit monoclonal antibody—8486S—Cell Signaling Technology) and anti-ALP (1:1000, rabbit monoclonal antibody—7074S—Abcam) and the secondary antibody goat anti-rabbit IgG-HPR (1:3000—7074S—Cell Signaling Technology) overnight at 4 °C and 1 h at room temperature, respectively. Beta-actin protein was used as control and detected with primary anti-beta-actin (1:1000; goat polyclonal antibody—sc1615—Santa Cruz Biotechnology, Dallas, TX, USA) and secondary antibody donkey anti-goat IgG-HPR (1:3000;—sc2003—Santa Cruz Biotechnology). The protein bands were detected using Clarity^TM^ Western ECL Substrate (Bio-Rad Laboratories). RUNX2 and ALP were quantified (n = 3) using ImageJ Software (NIH, Bethesda, MD, USA) and normalized to beta-actin protein expression.

### 2.4. Effect of MSCs^+BMP-9^ Primed with Hypoxia on Bone Repair of Rat Calvarial Defects

#### 2.4.1. Surgical Procedure to Create and Treatment of Calvarial Defects

All animal procedures were approved by the Committee of Ethics in Animal Research of the School of Dentistry of Ribeirão Preto, University of São Paulo (Protocol # 2021.1.15.58.2). Twenty-four Sprague Dawley rats weighing 180 g were anesthetized with ketamine (75 mg/kg, intraperitoneal; Agener União, Embu-Guaçu, SP, Brazil) and xylazine (6 mg/kg, intraperitoneal; Calier, Juatuba, MG, Brazil) to create a unilateral 5 mm diameter defect with a trephine drill (Neodent, Curitiba, PR, Brazil). After 2 weeks, the rats were randomly distributed (n = 12 per group) and anesthetized, and the calvarial defects were treated with a local injection of 5 × 10^6^ MSCs^+BMP-9^ cultured under either control or hypoxia condition in 50 µL of phosphate-buffered saline (PBS, Gibco-Life Technologies). One rat in the control group died after cell injection, and the morphometry analysis was performed on eleven animals of this group.

#### 2.4.2. Microtomographic (µCT) Analysis

The rats were anesthetized, and the µCT analysis was performed by a single-blinded researcher using the SkyScan 1276 system (Bruker–Skyscan, Kontich, Belgium) immediately after cell injection and 14 and 28 days post-cell injection. The three-dimensional reconstructions were created using NRecon Cluster software (Micro Photonics Inc., Allentown, PA, USA). The morphometric parameters, bone volume (BV), percentage of bone volume (BV/TV), bone surface (BS), trabecular thickness (Tb.Th), trabecular number (Tb.N) and bone mineral density (BMD), were measured in 5 mm diameters of the calvarial defects.

#### 2.4.3. Histological Analysis

After µCT analysis, the calvarias were harvested and fixed in 10% buffered formalin, decalcified in 4% ethylenediaminetetraacetic acid (Merck Millipore, Darmstadt, Hesse, Germany), washed and dehydrated in alcohol. The samples were embedded in paraffin (Sigma-Aldrich), cut with 5 μm thickness and stained with hematoxylin and eosin (Neon, Suzano, SP, Brazil). The images were analyzed and acquired using a light microscope Axioskop 40 (Carl Zeiss, Oberkochen, BW, Germany) coupled to a digital camera Axiocam Icc3 (Carl Zeiss).

### 2.5. Statistical Analyses

The data of cell viability (n = 5) and *Glut1* and *Vegfa* gene expression (n = 4) were compared by using one-way ANOVA followed by Tukey’s post-test. The data of HIF-1α protein expression (n = 3) were compared by using two-way ANOVA followed by Tukey’s post-test. All other data of protein amount and expression (n = 3), gene expression (n = 4), cell proliferation (n = 5), cell migration (n = 5) and bone morphometric parameters (n = 11 for control and n = 12 for hypoxia) were compared by using Student’s *t* test. The statistical analyses of the parameters evaluated over time (cell proliferation, cell migration and bone morphometry) were performed based on the area under the curve (Supporting Information, Table S2). The data were expressed as mean ± standard deviation (*p* ≤ 0.05).

## 3. Results

### 3.1. Hypoxia Induction and Duration of Its Effect on MSCs^+BMP-9^

**Figure 1:**
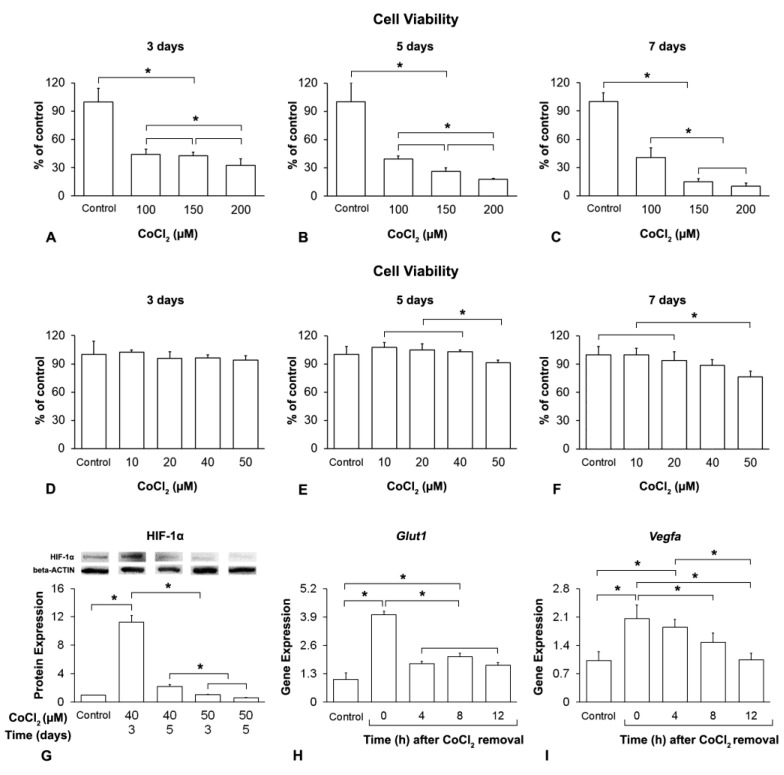
Hypoxia induction and duration of its effect on MSCs^+BMP-9^. Viability of MSCs^+BMP-9^ cultured in either absence (control) or presence of CoCl_2_ at 100, 150 and 200 μM for the last 3 (**A**), 5 (**B**) and 7 days (**C**) of culture and at 10, 20, 40 and 50 μM in the last 3 (**D**), 5 (**E**) and 7 days (**F**) of culture, measured on day 8. Protein expression of HIF-1α in MSCs^+BMP-9^ cultured in either absence (control) or presence of CoCl_2_ at 40 and 50 μM for the last 3 and 5 days, detected on day 8 (**G**). Gene expression of *Glut1* (**H**) and *Vegfa* (**I**) in MSCs^+BMP-9^ cultured for 8 days, with the last 3 days in either absence (control) or presence of CoCl_2_ at 40 μM, detected 0, 4, 8 and 12 h after CoCl_2_ removal. The data are presented as mean ± standard deviation, and the * indicates statistically significant differences (*p* ≤ 0.05).

The selection of the CoCl_2_ concentration and exposure time to induce hypoxia was based on the cell viability, HIF-1α protein expression and gene expression of the hypoxia targets, *Glut1* and *Vegfa* (Figure 1). The first set of experiments to evaluate MSCs^+BMP-9^ viability was carried out with concentrations of CoCl_2_ from 100 to 200 μM, which induced deleterious effects on viability when cells were treated from the last 3 days (Figure 1A–C). CoCl_2_ at 100, 150 and 200 μM decreased viability compared with control when cells were treated for the last 3 (Figure 1A), 5 (Figure 1B) and 7 days (Figure 1C) of culture (*p* < 0.001 for all concentrations and times of treatment). Cell viability was lower in the presence of CoCl_2_ at 200 compared with 100 μM when cells were treated for the last 3 (*p* = 0.038) and 5 days (*p* = 0.012), without a statistically significant difference between 150 and 100 μM (*p* = 0.991 and *p* = 0.174) and between 150 and 200 μM (*p* = 0.066 and *p* = 0.488, Figure 1A,B). Cell viability was lower in the presence of CoCl_2_ at 200 and 150 μM, without a statistically significant difference between them (*p* = 0.747) or compared with 100 μM (*p* < 0.001 for both) and with control (*p* < 0.001 for both), and it was lower at 100 μM compared with control (*p* < 0.001) when cells were treated for the last 7 days of culture (Figure 1C). In another set of experiments, MSCs^+BMP-9^ were treated with concentrations of CoCl_2_ from 10 to 50 μM, with 50 μM being the only concentration that exerted some deleterious effect on viability when cells were treated for at least the last 5 days of culture (Figure 1D–F). The viability was not affected (*p* = 0.371) by CoCl_2_ when cells were treated with concentrations from 10 to 50 μM for the last 3 days of culture (Figure 1D). CoCl_2_ at 50 μM decreased viability compared with 10 (*p* = 0.001), 20 (*p* = 0.005) and 40 μM (*p* = 0.018), without a statistically significant difference compared with control (*p* = 0.142) when cells were treated for the last 5 days of culture (Figure 1E). CoCl_2_ at 50 μM decreased viability compared with control (*p* = 0.001), 10 (*p* < 0.001) and 20 μM (*p* = 0.012), without a statistically significant difference compared with 40 μM (*p* = 0.117) when cells were treated for the last 7 days of culture (Figure 1F).

Based on these findings, the treatments with CoCl_2_ at 40 and 50 μM for the last 3 and 5 days of culture were selected to evaluate the HIF-1α protein expression in MSCs^+BMP-9^, which was higher at 40 μM for the last 3 days of culture compared with all other concentrations and times of treatment as well as with control (*p* < 0.001 for all comparisons; Figure 1G). The HIF-1α protein expression was higher in cells treated with CoCl_2_ at 40 μM for the last 5 days of culture compared with 50 μM for either the last 3 (*p* = 0.001) or 5 days (*p* < 0.001) of culture, without a statistically significant difference between them (*p* = 0.202, Figure 1G).

The next set of experiments was performed to confirm the hypoxia induction and to assess the lasting effect of CoCl_2_ treatment after its removal by evaluating the gene expression of the hypoxia targets *Glu1* and *Vegfa*. The assays were conducted with MSCs^+BMP-9^ treated with CoCl_2_ at 40 μM for the last 3 days of culture, which were defined as the parameters for mimicking hypoxia in this culture model. The *Glut1* gene expression was higher at the time of CoCl_2_ removal (0 h) compared with 4, 8 and 12 h post-CoCl_2_ removal and with control (*p* < 0.001 for all comparisons), and it was higher at 4 (*p* < 0.001), 8 (*p* < 0.001) and 12 h (*p* = 0.002) post-CoCl_2_ removal compared with control (Figure 1H). No statistically significant differences were observed between 4 and 8 h (*p* = 0.148), 4 and 12 h (*p* = 0.978) and 8 and 12 h (*p* = 0.054) post-CoCl_2_ removal (Figure 1H). The *Vegfa* gene expression was higher at the time of CoCl_2_ removal (0 h) compared with 8 (*p* = 0.024) and 12 h (*p* < 0.001) post-CoCl_2_ removal and with control (*p* < 0.001), and it was higher at 4 compared with 12 h post-CoCl_2_ removal (*p* = 0.002) and with control (*p* = 0.002, Figure 1I). No statistically significant differences were observed between 8 and 4 h (*p* = 0.221) and 8 and 12 h (*p* = 0.122) post-CoCl_2_ removal and between 8 h and control (*p* = 0.101) and 12 h and control (*p* = 1.00, Figure 1I).

### 3.2. Effect of Hypoxia on the Expression of Components and Targets of the BMP Signaling Pathway in MSCs^+BMP-9^

**Figure 2:**
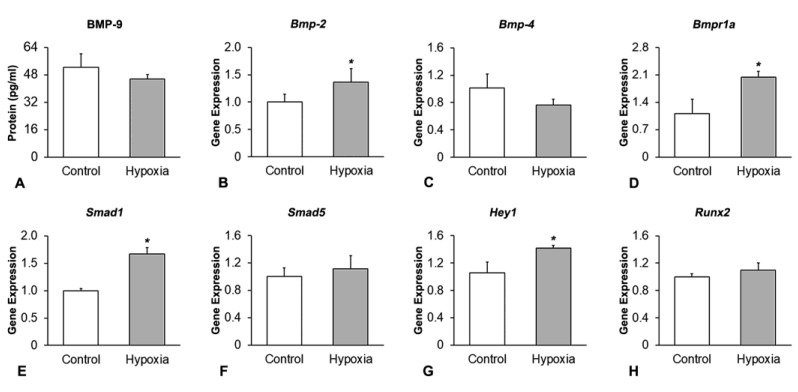
Effect of hypoxia on the expression of components and targets of the BMP signaling pathway in MSCs^+BMP-9^. Protein expression of BMP-9 (**A**), and gene expression of *Bmp-2* (**B**), *Bmp-4* (**C**), *Bmpr1a* (**D**), *Smad1* (**E**), *Smad5* (**F**), *Hey1* (**G**) and *Runx2* (**H**) in MSCs^+BMP-9^ cultured under either control or hypoxia condition, on day 8. The data are presented as mean ± standard deviation, and the * indicates statistically significant differences (*p* ≤ 0.05).

The effect of hypoxia on MSCs^+BMP-9^ was evaluated at the time of hypoxia removal (0 h), on day 8 (Figure 2). Hypoxia did not affect the amount of BMP-9 protein (*p* = 0.219, Figure 2A) and the gene expression of *Bmp-4* (*p* = 0.060, Figure 2C), *Smad5* (*p* = 0.369, Figure 2F) and *Runx2* (*p* = 0.140, Figure 2H) compared with control. The gene expression of *Bmp*-2 (*p* = 0.029, Figure 2B), *Bmpr1a* (*p* = 0.003, Figure 2D)*, Smad1* (*p* < 0.001, Figure 2E) and *Hey1* (*p* = 0.018, Figure 2G) was upregulated by hypoxia compared with control.

### 3.3. Effect of CM of MSCs^+BMP-9^ Primed with Hypoxia on Cell Proliferation, Migration and Osteoblast Differentiation of MSCs

**Figure 3:**
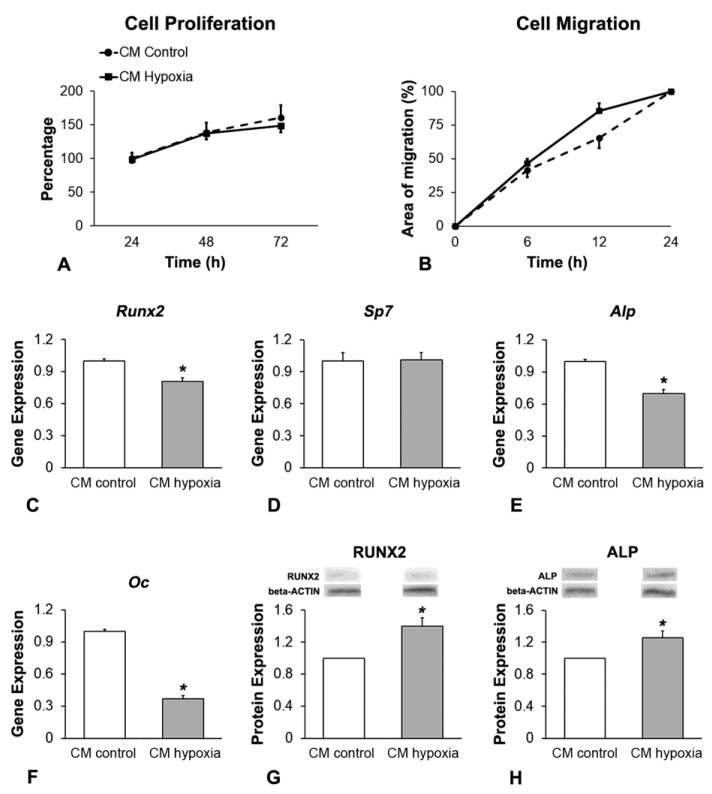
Effect of CM of MSCs^+BMP-9^ primed with hypoxia on cell proliferation, migration and osteoblast differentiation of MSCs. Cell proliferation at 24, 48 and 72 h (**A**) and migration at 0, 6, 12 and 24 h (**B**) of MSCs cultured in CM of MSCs^+BMP-9^ cultured under either control (CM control) or hypoxia condition (CM hypoxia). Gene expression of *Runx2* (**C**), *Sp7* (**D**), *Alp* (**E**) and *Oc* (**F**) and protein expression of RUNX2 (**G**) and ALP (**H**) of MSCs cultured either in CM control or CM hypoxia, on day 7. The data are presented as mean ± standard deviation, and the * indicates statistically significant differences (*p* ≤ 0.05).

Based on the duration of the effect of CoCl_2_, the MSCs^+BMP-9^-derived CM was collected 4 h post-hypoxia removal and used to treat immortalized MSCs (Figure 3). Cell proliferation was not affected (*p* = 0.414) by CM hypoxia compared with CM control (Figure 3A). CM hypoxia promoted more cell migration (*p* = 0.006) compared with CM control (Figure 3B). The gene expression of *Runx2* (Figure 3C), *Alp* (Figure 3E) and *Oc* (Figure 3F) was downregulated (*p* < 0.001 for all genes), whereas *Sp7* (Figure 3D) was not affected (*p* = 0.856) by CM hypoxia compared with CM control on day 7. The protein expression of RUNX2 (*p* = 0.003, Figure 3G) and ALP (*p* = 0.006, Figure 3H) was upregulated by CM hypoxia compared with CM control on day 7.

### 3.4. Effect of CM of MSCs^+BMP-9^ Primed with Hypoxia on Cell Proliferation, Migration and Osteoblast Differentiation of MC3T3-E1 Cells

**Figure 4:**
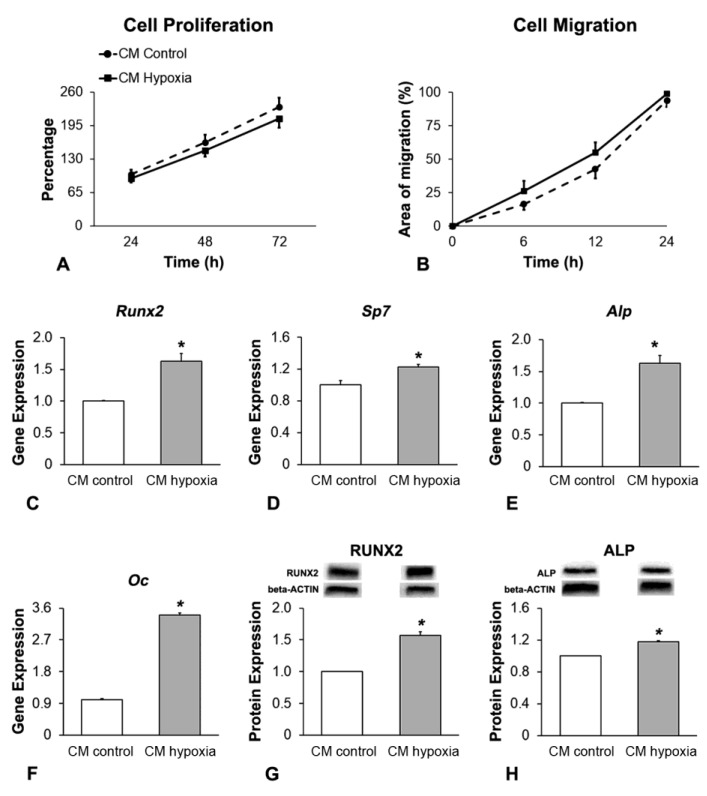
Effect of CM of MSCs^+BMP-9^ primed with hypoxia on cell proliferation, migration and osteoblast differentiation of MC3T3-E1 cells. Cell proliferation at 24, 48 and 72 h (**A**) and migration at 0, 6, 12 and 24 h (**B**) of MC3T3-E1 cells cultured in CM of MSCs^+BMP-9^ cultured under either control (CM control) or hypoxia condition (CM hypoxia). Gene expression of *Runx2* (**C**), *Sp7* (**D**), *Alp* (**E**) and *Oc* (**F**) and protein expression of RUNX2 (**G**) and ALP (**H**) of MC3T3-E1 cells cultured either in CM control or CM hypoxia, on day 7. The data are presented as mean ± standard deviation, and the * indicates statistically significant differences (*p* ≤ 0.05).

The MSCs^+BMP-9^-derived CM was collected 4 h post-hypoxia removal and used to treat MC3T3-E1 cells (Figure 3). Cell proliferation was not affected (*p* = 0.291) by CM hypoxia compared with CM control (Figure 4A). CM hypoxia promoted more cell migration (*p* = 0.011) compared with CM control (Figure 4B). The gene expression of *Runx2* (Figure 4C), *Sp7* (Figure 4D), *Alp* (Figure 4E) and *Oc* (Figure 4F) was upregulated (*p* < 0.001 for all genes), as well as the protein expression of RUNX2 (*p* = 0.003, Figure 4G) and ALP (*p* = 0.006, Figure 4H), by CM hypoxia compared with CM control on day 7.

### 3.5. Effect of MSCs^+BMP-9^ Primed with Hypoxia on Bone Repair of Rat Calvarial Defects

**Figure 5:**
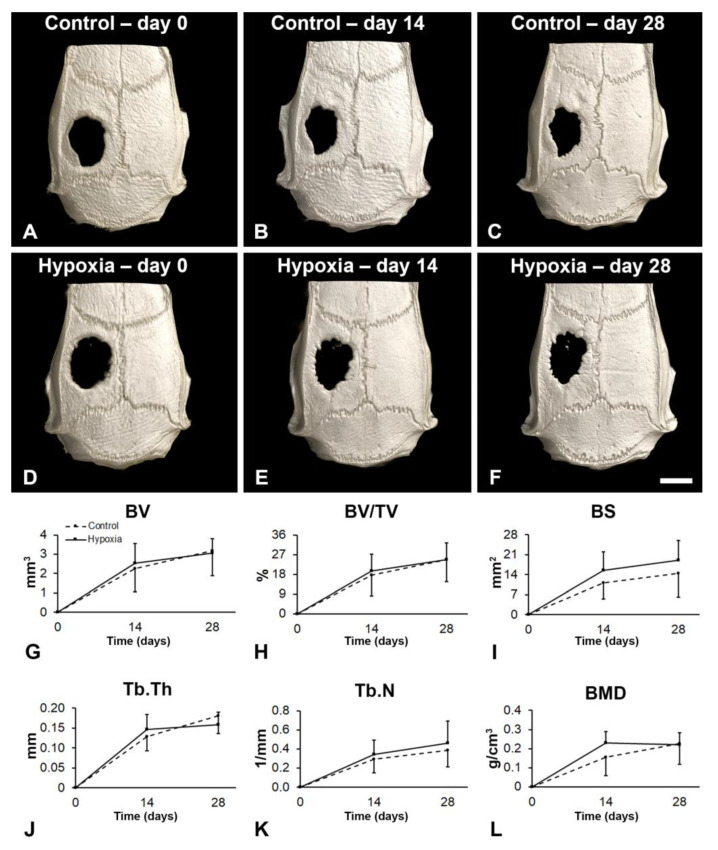
Effect of MSCs^+BMP-9^ primed with hypoxia on bone repair of rat calvarial defects. Analysis of the bone tissue by µCT. Three-dimensional reconstructions of rat calvarial defects treated with either control MSCs^+BMP-9^ (Control, (**A**–**C**)) or MSCs^+BMP-9^ primed with hypoxia (Hypoxia, (**D**–**F**)) locally injected 2 weeks post-defect creation (day 0) and evaluated at 14 and 28 days post-treatment. Morphometric parameters bone volume (BV, (**G**)), percentage of bone volume (BV/TV, (**H**)), bone surface (BS, (**I**)), trabecular thickness (Tb.Th, (**J**)) trabecular number (Tb.N, (**K**)) and bone mineral density (BMD, (**L**)) evaluated in the region of interest, within the 5 mm diameter of the calvarial defect. The data are presented as mean ± standard deviation (n = 11 for control and n = 12 for hypoxia). Scale bar: (**A**–**F**) = 3 mm.

Bone repair was induced by injecting MSCs^+BMP-9^ cultured under either control or hypoxia condition into rat calvarial bone defects (Figure 5). The three-dimensional reconstructions indicated that the bone formation increased over time in both defects injected with control MSCs^+BMP-9^ (Figure 5A–C) and hypoxia-primed MSCs^+BMP-9^ (Figure 5D–F). Additionally, the bone repair induced by MSCs^+BMP-9^ primed with hypoxia (Figure 5B,C) was similar to control MSCs^+BMP-9^ (Figure 5E,F), and the morphometric parameters confirmed this finding. MSCs^+BMP-9^ primed with hypoxia did not increase the BV (*p* = 0.748, Figure 5G), BV/TV (*p* = 0.720, Figure 5H), BS (*p* = 0.114, Figure 5I), Tb.Th (*p* = 0.780, Figure 5J), Tb.N (*p* = 0.395, Figure 5K) and BMD (*p* = 0.160, Figure 5L) compared with control MSCs^+BMP-9^.

**Figure 6:**
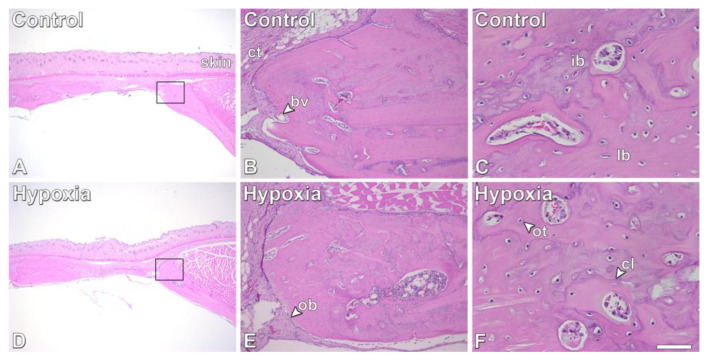
Effect of MSCs^+BMP-9^ primed with hypoxia on bone repair of rat calvarial defects. Histological analysis of the bone tissue. Micrographs of rat calvarial defects treated with either control MSCs^+BMP-9^ (Control, (**A**–**C**)) or MSCs^+BMP-9^ primed with hypoxia (Hypoxia, (**D**–**F**)) locally injected 2 weeks post-defect creation and evaluated at 28 days post-treatment. The square in (**A**) is presented in (**B**), and the square in (**D**) is presented in (**E**). Scale bar: (**A**,**D**) = 1.25 mm; (**B**,**E**) = 200 μm; (**C**,**F**) = 50 μm. bv: blood vessel; cl: cement line; ct: connective tissue; ib: immature bone; lb: lamellar bone; ob: osteoblast; ot: osteocyte.

The histological sections corroborated the µCT analysis (Figure 6). No significant differences in terms of bone tissue features were observed between defects injected with either control MSCs^+BMP-9^ (Figure 6A–C) or hypoxia-primed MSCs^+BMP-9^ (Figure 6D–F). The newly formed bone exhibited characteristics of healthy tissue with areas of immature and lamellar bone, and the presence of osteoblasts, osteocytes and blood vessels, without signs of adverse reactions irrespective of treatment.

## 4. Discussion

The osteogenesis is controlled by a wide web of signaling molecules including BMPs and HIFs, and among them, the BMP-9-HIF-1α circuit plays a relevant role in osteoblast differentiation. Here, by using CoCl_2_ as a hypoxia inductor, it was demonstrated that hypoxia modulated the expression of BMP components and targets in MSCs^+BMP-9^ without affecting the amount of BMP-9 protein and that CM of hypoxia-primed MSCs^+BMP-9^ enhanced cell migration and osteoblast differentiation of MSCs and MC3T3-E1. Additionally, the results indicated that hypoxia-primed MSCs^+BMP-9^ and control MSCs^+BMP-9^ induced similar bone repair in rat calvarial defects.

Prior to investigating the effect of hypoxia on osteoblastic cell responses, we determined the efficient concentration and time of exposure to CoCl_2_ to mimic a hypoxic microenvironment in the MSCs^+BMP-9^ culture model. The CoCl_2_ is considered a hypoxia simulant because it prevents HIF-1α degradation, despite different outcomes that have been reported when the effects of actual hypoxia are compared with CoCl_2_, with hypoxia exhibiting more pronounced effects than CoCl_2_ on osteoblasts [33,34]. This finding could be related to the fact that oxygen availability may affect cellular mechanisms other than HIF-1α stabilization [34]. However, as one of the main effects of hypoxia is the inhibition of HIF-1α degradation, CoCl_2_ is an appropriate tool to reproduce an in vitro hypoxic microenvironment [35,36]. It was demonstrated that CoCl_2_ at 50 and 100 µM significantly reduces the viability of MSCs from mouse bone marrow [37]. Here, we tested concentrations from 10 to 200 µM and observed a dramatic reduction in cell viability induced by concentrations equal to or greater than 100 µM. Thus, only the effect of CoCl_2_ at 40 and 50 µM on HIF-1α expression was evaluated and confirmed that the treatment with 40 µM for the last 3 days of culture induced the higher expression of HIF-1α, which is within the range used in other studies that evaluated the same parameter in similar culture models [37,38]. It was also investigated for how long the effect of CoCl_2_ lasts after the stimulus removal by evaluating the gene expression of two HIF-1α downstream factors, *Glut1*, the most common glucose transporter, and *Vegfa*, a crucial molecule for angiogenesis [39]. The expression of *Glut1* and *Vegfa* peaked at the time of CoCl_2_ withdrawal, but *Glut1* was still higher than in the control up to 12 h and *Vegfa* 8 h after CoCl_2_ removal. It was observed that the effect of CoCl_2_ on HIF-1α expression was reversed 2 h after stimulus withdrawal, but it is possible that this transitory HIF-1α upregulation induced by CoCl_2_ impacts the expression of downstream targets for longer periods as we demonstrated in this study [37].

BMP-9 increases the HIF-1α expression, and the downregulation of HIF-1α inhibits osteoblast differentiation induced by BMP-9, a mechanism that is regulated by Runx2, a downstream target of HIF-1α [27,28,33]. Based on this cellular mechanism, we worked with MSCs^+BMP-9^, an immortalized cell line that expresses BMP-9, which enhances their ability to induce bone repair [21]. Interestingly, the hypoxia model used here, which increased the HIF-1α expression, did not affect the amount of BMP-9 but upregulated the expression of *Bmp-2*, *Bmpr1a*, *Smad1* and *Hey1*, all components or targets of the BMP signaling pathway. Specifically, Hey1 is upregulated by BMP-2 when MSCs are differentiated into osteoblasts, and it is a direct target of BMP-9 signaling being upregulated at the early stages of osteoblast differentiation [40,41]. Thus, despite the lack of effect of hypoxia on BMP-9 expression, it is not possible to discard its participation in the effect of hypoxia on osteoblast differentiation, as well as other BMPs such as BMP-2, since hypoxia upregulated several components of the BMP signaling, including *Hey1*.

Considering cell therapy approaches, the paracrine effects of cells used to treat bone defects may favor bone formation by acting on host cells to modulate inflammation, immune response and angiogenesis [42]. Thus, we evaluated the paracrine effects of MSCs^+BMP-9^ primed with hypoxia on MSCs and MC3T3-E1 cells by using their conditioned medium. In general, this conditioned medium did not affect viability but increased migration of both cell lineages. The different results in terms of *Runx2*, *Alp* and *Oc* gene expression in MSCs and MC3T3-E1 suggest that the regulation of the mRNA expression of bone markers by hypoxia depends on the stage of osteoblast differentiation. Yet, the positive effect of hypoxia on osteoblast phenotype markers, RUNX2 and ALP protein expression, was similar irrespective of cell type and culture condition, indicating the stimulant effect of the association of hypoxia and BMP-9 on the osteogenic potential of these cells. Such findings are due to the molecules secreted by MSCs^+BMP-9^ primed with hypoxia, since they were noticed in cultures grown in the conditioned medium generated under hypoxia. Corroborating our findings, despite not using a conditioned medium but evaluating cells under a hypoxic condition, several studies have shown the upregulation of osteoblast differentiation induced by CoCl_2_, the hypoxia simulant used here [36,43,44]. Such an effect involves the signal transducer and activator of transcription 3, a transcription factor that mediates cell survival, proliferation and differentiation, and the zinc finger protein, FOG family member 2 [36,44].

The increased bone formation induced by MSCs^+BMP-9^ and the positive paracrine effects of MSCs^+BMP-9^ primed with hypoxia on cell migration and osteoblast differentiation drove the investigation on the bone repair induced by hypoxia-primed MSCs^+BMP-9^ in rat calvarial defects [21]. Despite observing a continuous increase in bone formation along the 28-day period of observation, hypoxia failed to stimulate the bone repair promoted by MSCs^+BMP-9^. The discrepancy between the in vitro and in vivo findings may be related to the different experimental approaches, since while cultured MSCs were treated with conditioned medium of MSCs^+BMP-9^ primed with hypoxia, the calvarial defects were treated with a local injection of MSCs^+BMP-9^ cultured under hypoxia condition. Thus, further investigations should be performed to find the ideal condition to favor bone repair, e.g., the use of conditioned medium of MSCs^+BMP-9^ primed with hypoxia to treat the bone defects instead of the MSCs^+BMP-9^ themselves cultured under hypoxia condition. Another limitation of this study is the lack of validation of the results in human cells, which should be investigated considering the potential application of this therapy in clinical situations. In contrast with our results, hypoxia-preconditioned human MSCs in 1% O_2_ for 3 days favored femoral fracture healing in a rat model [45]. Such different outcomes may be related to differences in the experimental designs such as the method to induce hypoxia, the bone defect model and the overexpression of BMP-9. Additionally, it is possible that the positive effect of hypoxia on bone repair induced by MSCs was surpassed by BMP-9 overexpression since we previously demonstrated that BV/TV of MSCs^+BMP-9^ was almost two-fold higher than the control MSCs [21].

## 5. Conclusions

It was demonstrated that hypoxia induced by CoCl_2_ regulates the expression of BMP signaling pathway components and targets in MSCs^+BMP-9^ without affecting the amount of BMP-9. Additionally, MSCs^+BMP-9^ primed with hypoxia enhanced cell migration and osteoblast differentiation via paracrine actions and due to, at least in part, the activation of the BMP-9-HIF-1α circuit. Thus, despite hypoxia having no effect on the osteogenic function of MSCs^+BMP-9^, these results open windows for further investigations on approaches to modulate the BMP-9-HIF-1α circuit in the context of cell-based therapies to induce bone regeneration.

## Data Availability

The data are available upon request from the authors.

## Supplementary Materials

The following supporting information can be downloaded at: https://www.mdpi.com/article/10.3390/biology12081147/s1, Table S1. Primer sequences or TaqMan assay identifications and product sizes (bp) for real-time polymerase chain reaction; Table S2. Area under the curve (arbitrary unit) calculated to carry out the statistical analyses of the parameters evaluated over time (cell proliferation, cell migration and bone morphometry). The data are presented as mean ± standard deviation and compared by using Student’s *t* test (*p* ≤ 0.05).
