# Supplementary material for: Effect of Mesenchymal Stem Cells Overexpressing BMP-9 Primed with Hypoxia on BMP Targets, Osteoblast Differentiation and Bone Repair
Source: Biology (Basel). 2023 Aug 19;12(8):1147. doi: 10.3390/biology12081147 (PMC10452403; doi:10.3390/biology12081147)
Supplement: Supplementary file 1 [file biology-12-01147-s001.zip › biology-2539598-supplementary.pdf]

Table S1. Primer sequences or TaqMan assay identifications and product sizes (bp) for real-time polymerase chain reaction

**Gene**	**Sense / anti-sense sequence or TaqMan Assay Identification**	**bp**
Glut1	CTG TCG GGT ATC AAT GCT GTG T<br>CGT CCA GCT CGC TCT ACA AC	149
Vegfa	AGC AGA TGT GAA TGC AGA CCA<br>AGG ATT TAA ACC GGG ATT TCT TGC	112
Bmp-2	Mm01962382_s1	101
Bmp-4	Mm03413826_mH	110
Bmpr1a	TGG GAG TGG ATC TGG ATT GC<br>TCA CCA CGC CAT TTA CCC AT	120
Smad1	GCT TCG TGA AGG GTT GGG G<br>GCT TCG TGA AGG GTT GGG G	147
Smad5	TTG TTC AGA GTA GGA ACT GCA AC<br>GAA GCT GAG CAA ACT CCT GAT	114
Hey1	CCG ACG AGA CCG AAT CAA TAA C<br>TCA GGT GAT CCA CAG TCA TCT G	125
Runx2	CTT CAC AAA TCC TCC CCA AGT G<br>GGA ATG CGC CCT AAA TCA CTG	150
Sp7	TTC TGC GGC AAG AGG TTC AC<br>TTG CTC AAG TGG TCG CTT CT	125
Alpl	GGG GTA CAA GGC TAG ATG GC<br>CGG GCT CAA AGA GAC CTA AGA	150
Oc	TTC TGC TCA CTC TGC TGA CC<br>GCT TGG ACA TGA AGG CTT TGT	99
Eif2b1	ACC TCC CTG GAA TAC TCT GAC T<br>TCG CCC CGT CTT TGA TGA AT	136
Eif2b1	Mm00460997_m1	83

Glut1: solute carrier family 2 (facilitated glucose transporter), member 1, *Vegfa*: vascular endothelial growth factor A, *Bmp-2*: bone morphogenetic protein 2, *Bmp-4*: bone morphogenetic protein 4, *Bmpr1a*: bone morphogenetic protein receptor, type 1A, *Smad1*: SMAD family member 1, *Smad5*: SMAD family member 5, *Hey1*: hairy/enhancer-of-split related with YRPW motif 1, *Runx2*: runt-related transcription factor 2, *Sp7*: osterix, *Alpl*: alkaline phosphatase, *Oc*: osteocalcin, *Eif2b1*: eukaryotic translation initiation factor 2B subunit 1 (alpha)

Table S2. Area under the curve (arbitrary unit) calculated to carry out the statistical analyses of the parameters evaluated over time (cell proliferation, cell migration and bone morphometry). The data are presented as mean ± standard deviation and compared by Student's t test ($p \leq 0.05$)

**Parameter**	**Control**	**Hypoxia**	**p-value**
MSC proliferation	269.25 ± 20.04	260.56 ± 10.36	0.414
MSC migration	239.96 ± 16.63	274.93 ± 11.70	0.006
MC3T3-E1 proliferation	327.77 ± 29.13	297.38 ± 52.25	0.291
MC3T3-E1 migration	174.42 ± 18.63	207.76 ± 13.34	0.011
BV	3.85 ± 1.81	4.07 ± 1.31	0.748
BV/TV	30.05 ± 14.01	31.97± 10.55	0.720
BS	18.38 ± 9.61	25.08 ± 9.41	0.114
Tb.Th	0.22 ± 0.05	0.23 ± 0.05	0.780
Tb.N	0.49 ± 0.22	0.57 ± 0.25	0.395
BMD	0.27 ± 0.14	0.34 ± 0.09	0.160

MSC: mesenchymal stem cell, BV: bone volume, BV/TV: percentage of bone volume, BS: bone surface, Tb.Th: trabecular thickness, Tb.N: trabecular number, BMD: bone mineral density
